# Interatrial Block as a Common Finding in Patients with Acute Pulmonary Artery Embolism

**DOI:** 10.3390/jcm14238405

**Published:** 2025-11-27

**Authors:** Fabienne Kreimer, Tobias J. Brix, Felix K. Wegner, Dennis Korthals, Hilke Könemann, Florian Doldi, Julian Wolfes, Antonius Büscher, Christian Ellermann, Julia Köbe, Florian Reinke, Benjamin Rath, Fatih Güner, Gerrit Frommeyer, Lars Eckardt, Michael Gotzmann

**Affiliations:** 1 Department of Cardiology II—Rhythmology, University Hospital Münster, 48149 Münster, Germany; 2 Institute of Medical Informatics, University of Münster, 48149 Münster, Germany; 3 Department of Cardiology and Rhythmology, University Hospital St. Josef Hospital Bochum, 44791 Bochum, Germany

**Keywords:** pulmonary artery embolism, ECG, interatrial block

## Abstract

**Background/Objectives:** Electrocardiographic (ECG) findings such as sinus tachycardia and right bundle branch block are commonly associated with acute pulmonary embolism (PE). This study aimed to investigate the prevalence of advanced interatrial block (IAB) in patients with acute PE and its association with atrial fibrillation (AF) and hemodynamic changes. **Methods:** This retrospective, single-center study included patients diagnosed with acute PE (42% female, 58% male) between January 2014 and September 2024 at University Hospital Münster. A control group of individuals without manifest heart disease (45% female, 55% male) served as a control group. All patients underwent clinical, laboratory, ECG, and echocardiographic evaluations. **Results:** A total of 351 patients with acute PE and 120 control patients were included. The PE group had a mean age of 62.5 years. Advanced IAB was detected in 35% of PE patients, significantly higher than in controls (2%). In contrast, typical ECG signs of PE such as sinus tachycardia (23%), right bundle branch block (8%), and S1Q3 pattern (20%) were less frequent. A subgroup analysis demonstrated that patients with IAB were older, had a higher CHA_2_DS_2_-VA score, and were more likely to have pre-existing and new-onset AF. IAB was not associated with right heart dysfunction on echocardiography. **Conclusion:** For the first time, this study revealed that advanced IAB was present in many patients with acute PE. IAB was associated with a higher risk of AF and greater thromboembolic risk but not with hemodynamic changes typical of PE. Detecting an advanced IAB at the initial presentation in the emergency department could provide an important indication of PE.

## 1. Introduction

Acute pulmonary embolism (PE) is a life-threatening condition, often leading to increased volume and pressure load of the right heart [1]. The pathophysiologic alterations resulting from PE can manifest in several ways, including changes in cardiac hemodynamics, electrophysiologic abnormalities, and the occurrence of arrhythmias [2]. Electrocardiographic (ECG) findings such as sinus tachycardia, right bundle branch block and SI/QIII-pattern are often associated with PE and reflect the underlying cardiac dysfunction [3,4]. An interatrial block (IAB), a conduction abnormality that impairs the interatrial electrical impulse propagation, has been associated with an increased risk of atrial fibrillation (AF), a common arrhythmia in PE patients [5,6,7]. Therefore, the aim of this study was to investigate the prevalence of advanced IAB in patients with acute PE and to compare it with a control group of individuals without manifest heart disease. In addition, the association between IAB and other clinical factors such as AF, comorbidities and hemodynamic changes was investigated to better understand the potential role of IAB in the pathophysiology of PE.

## 2. Methods

In the present cohort study, all patients from January 2014 to September 2024 who were treated for acute PE at University Hospital Münster or who suffered an acute PE during hospitalization were examined. Clinical parameters, laboratory parameters, ECG and echocardiographic findings were analyzed in all patients. Patients without manifest heart disease who were prospectively included served as a control group. The healthy control group has been enrolled prospectively since 2023. The local ethics committee approved the study (registration number 22-7535).

Inclusion criteria were the diagnosis of acute PE in the period from January 2014 to September 2024. Patients were excluded if no ECG was available or if the PE was only an incidental finding on chest CT or pulmonary scintigraphy without clinical symptoms or evidence of hemodynamic relevance. All patients without sinus rhythm on ECG were also excluded to enable P-wave analysis. The study period was limited to the duration of hospitalization. Patients with PE were compared with the control group regarding clinical, ECG, and echocardiographic findings.

**Figure 1:**
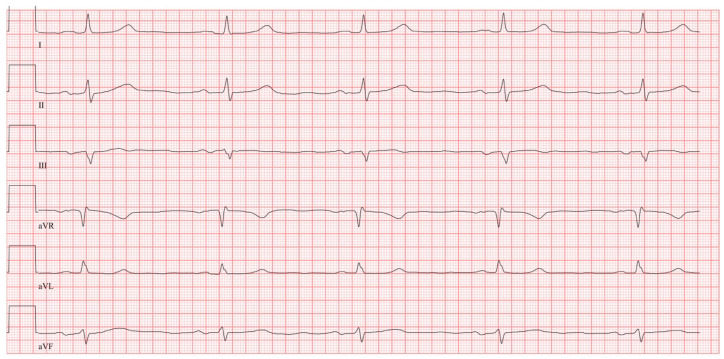
Example of an advanced interatrial block in a patient with acute pulmonary embolism. ECG was recorded at a rate of 50 mm/s and a voltage of 10 mm/mV.

A complete analysis of the 12-lead ECG was performed in all patients. The standard 12-lead surface ECG was recorded at a rate of 50 mm/s and a voltage of 10 mm/mV. All analyses were conducted by a single observer who was blinded to the patient group (PE group vs. control group). The ECG analysis included, in particular, P-wave indices but also an analysis of the QRS complex. The SI/QIII pattern was defined as a deep S-wave in lead I in combination with a Q-wave in lead III. An advanced IAB was present with a P-wave duration of a minimum of 120 ms and a biphasic morphology in leads II, III and aVF, but at least in III and aVF (Figure 1) [7]. A P dextroatriale was defined if the amplitude of the P-wave was above 0.25 mV.

The software SPSS 29.0 was used to perform statistical analyses. The numerical values are presented as mean ± standard deviation. Continuous variables were compared between groups using an unpaired t-test (for normally distributed variables) or a Mann–Whitney U-test (for non-normally distributed variables). Chi-square analysis or Fisher’s exact test was used to compare categorical variables. Binary logistic regression analysis was used to identify the independent factors for differentiating patients with acute PE and patients without acute PE. This analysis included gender and the CHADS-VA score (heart failure, hypertension, age, diabetes, stroke, vascular disease) as well as electrocardiography parameters. The risk for the presence of PE was determined for the ECG parameters. The results are given as odds ratios. A *p*-value < 0.05 was considered significant. All probability values reported are two-sided.

## 3. Results

The study cohort included a total of 351 patients (42% female, 58% male) who suffered an acute PE. A group of 120 patients (45% female, 55% male) without manifest heart disease served as a control group. The PE group had a mean age of 62.5 years. Common comorbidities included arterial hypertension (56%), heart failure (34%), and coronary artery disease (20%). AF was present in 19% of PE patients, 8% had new-onset AF.

**Figure 2:**
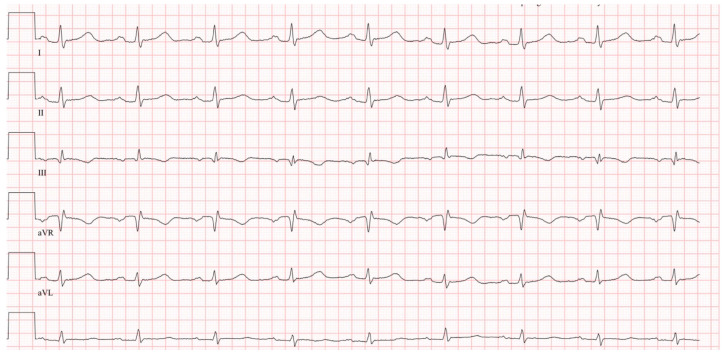
Example of a S1Q3 pattern in a patient with acute pulmonary embolism. ECG was recorded at a rate of 50 mm/s and a voltage of 10 mm/mV.

**Figure 3:**
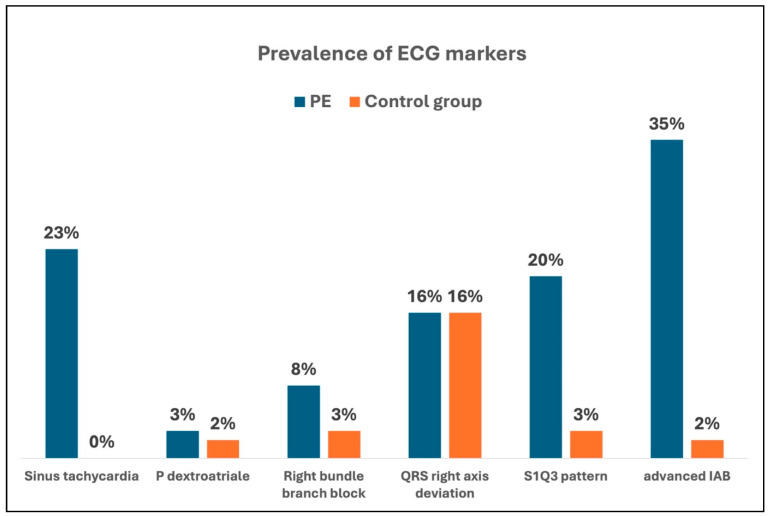
Prevalences of ECG markers of acute pulmonary embolism in the different groups. PE, pulmonary embolism; IAB, interatrial block.

Compared to the control group, patients with PE had a significantly higher CHA_2_DS_2_-VA score (3.2 vs. 1.6 pts., *p* < 0.001). Notably, the control group was older (62.5 vs. 67.9 years, *p* = 0.002). Except for arterial hypertension, patients with PE were significantly more likely to have heart failure, diabetes mellitus, stroke, peripheral arterial disease, AF and coronary artery disease. Patients with PE demonstrated significantly higher creatinine (1.2 vs. 0.9 mg/dL, *p* = 0.002), NT-proBNP (3383 vs. 224 pg/mL, *p* < 0.001), CRP (5.5 vs. 0.7 mg/dL, *p* < 0.001), and TSH (2.8 vs. 1.4 mU/L, *p* = 0.019) levels and significantly lower hemoglobin levels (12.4 vs. 13.9 g/dL, *p* < 0.001) than the control group. On echocardiography, patients with PE presented a mean left ventricular ejection fraction of 56%, which did not differ from the control group (56% vs. 57%, *p* = 0.131). In contrast, a significantly higher mean maximum left atrial volume (55 vs. 46 mL, *p* = 0.004), right atrial volume index (24 vs. 17 mL/m^2^, *p* < 0.001), and a larger right ventricular diameter (39 vs. 30 mm, *p* < 0.001) were detected in patients with PE. With a mean of 22 mm, TAPSE (tricuspid annular plane systolic excursion) values were lower than that of the control group (28 mm, *p* < 0.001). Pulmonary hypertension was more common in the PE group, as evidenced by higher mean systolic pulmonary artery pressures (35 vs. 17 mmHg, *p* < 0.001). Mitral valve regurgitation was more frequent in PE patients, though tricuspid regurgitation did not differ between groups. ECG revealed sinus tachycardia in 23% of patients with PE. Approximately 8% of patients had a right bundle branch block, 16% a QRS axis right deviation and 20% presented with an SI/QIII pattern (Figure 2). The PR interval, P-wave duration, P-wave axis and the prevalence of a P-dextroatriale did not differ from the control group, but in patients with PE, an advanced IAB was diagnosed in more than one third of patients (35%). Among the control group, an IAB occurred in only 2% of patients (*p* < 0.001). An advanced IAB occurred even more often in the presence of sinus tachycardia, QRS axis right deviation, or an SI/QIII pattern (Table 1, Figure 3). Binary logistic regression analysis showed that the parameters heart rate, IAB, S1/Q3 type, and right bundle branch block were independent ECG parameters for the diagnosis of acute PE.

The odds ratios (OR) of the ECG parameters are presented in Table 2. In the multivariate analysis, independent ECG markers for the presence of PE were heart rate (OR 1.081, confidence interval (CI) 1.054–1.108, *p* < 0.001), advanced IAB (OR 88.912, CI 18.258–432.986, *p* < 0.001), SI/QIII pattern (OR 5.150, CI 1.581–16.775, *p* = 0.007), and right bundle branch block (OR 6.891, CI 1.612–29.463, *p* = 0.009).

A subgroup analysis of PE patients with and without advanced IAB demonstrated that patients with IAB were significantly older (69.7 vs. 58.6 years, *p* < 0.001) and had a greater CHA_2_DS_2_-VA score (4.3 vs. 2.7 points, *p* < 0.001). Patients with IAB were more likely to suffer from arterial hypertension (69% vs. 48%, *p* < 0.001), coronary artery disease (30% vs. 15%, *p* = 0.002) and pre-existing AF (30% vs. 14%, *p* < 0.001). However, patients with IAB were also more frequently diagnosed with new-onset AF (16% vs. 4%, *p* < 0.001). On echocardiography, patients with IAB exhibited a larger left atrial volume index (32 vs. 27 mL/m^2^, *p* = 0.033) and more frequent mitral and tricuspid valve regurgitation than patients without IAB. The presence of IAB was not significantly associated with echocardiographic evidence of increased right heart dysfunction. Among patients with IAB, a lower heart rate (83 vs. 91 beats per minute, *p* < 0.001), longer PR interval (196 vs. 164 ms, *p* < 0.001 and P-wave duration (131 vs. 112 ms, *p* < 0.001), as well as leftward P-wave axis deviation (44 vs. 54°, *p* < 0.001) were more common. In contrast, a QRS axis rightward deviation occurred more frequently in patients without IAB (8% vs. 21%, *p* = 0.003) (Table 3).

## 4. Discussion

The present study investigated patients with acute PE and compared them to a control group without manifest heart disease. Key findings include:

(1) Advanced IAB was detected in 35% of PE patients, more frequently than other typical PE ECG signs like sinus tachycardia.
(2) An advanced IAB was by far the most important ECG parameter for the diagnosis of PE.
(3) Patients with IAB were older, had more comorbidities, and were more likely to develop new-onset AF during hospitalization, but IAB was not associated with altered PE related hemodynamics.

A PE is often associated with altered cardiac hemodynamics and right heart dysfunction, which can be reflected in the ECG [1]. ECG phenomena that are commonly seen in PE include sinus tachycardia, SI/QIII pattern, right bundle branch block and T wave inversions in the right precordial leads [1]. The presence of these ECG signs often indicates a hemodynamic relevance of the PE [4,8]. In our study, sinus tachycardia was detected in 23% of patients with PE. In addition, 8% of patients had right bundle branch block, 16% had a QRS axis deviation to the right and 20% exhibited an SI/QIII pattern. These findings are consistent with previous studies analyzing the prevalence of ECG signs in acute PE [3,9]. The present study investigated for the first time the prevalence of advanced IAB in a cohort of patients with acute PE. The prevalence was found to be notably high at 35%, which was higher than that of other typical ECG signs associated with PE. This high prevalence is even more remarkable when compared to the prevalence in a cardiac healthy, even slightly older cohort. One may suggest that alterations in right heart hemodynamics, including elevated pressures and volume overload, may trigger the acute onset of an IAB or reveal a potentially previously latent interatrial conduction delay. To our knowledge, no previous study has systematically evaluated advanced IAB in the setting of acute PE, making the present analysis the first to draw attention to this potential electrophysiological marker. The unexpectedly high prevalence of advanced IAB therefore raises the possibility that acute PE may unmask underlying atrial vulnerability or interact with pre-existing atrial cardiomyopathic changes and endothelial dysfunction.

Recent studies have indicated a significant association between advanced IAB and AF, since the presence of advanced IAB is considered a potential marker for an increased risk of AF [5,6,10,11]. Additionally, structural and functional atrial changes that contribute to IAB, such as atrial dilation or fibrosis, may further predispose to AF [6]. In the present study, new-onset AF was diagnosed in 8% of patients with PE, a rate consistent with previous studies [2,9]. Of note, when comparing patients with and without advanced IAB, the incidence of AF was significantly higher in patients with IAB (16% vs. 4%). A significant difference was also observed in the prevalence of pre-existing AF. Among patients with IAB, 30% had a history of AF prior to the PE, compared to only 14% in patients without IAB. These findings may indicate that advanced IAB serves as an ECG marker of a pre-existing atrial substrate prone to arrhythmogenesis, which becomes clinically relevant in the context of acute cardiorespiratory stress such as PE.

AF is often associated with PE, with new-onset AF observed in a significant proportion of PE patients [2]. The development of AF associated with PE can be attributed to several factors, including acute hemodynamic stress and right heart dysfunction caused by the embolism [1]. These underlying conditions can alter atrial conduction and increase the likelihood of cardiac arrhythmias such as AF. The issue of whether AF can be a cause of PE is more complex [12]. Although AF itself is a risk factor for thromboembolism due to the increased risk of clot formation, it is not usually considered a direct cause of PE [13,14]. In theory, however, embolisms originating from right atrial thrombi in patients with AF can lead to PE [12,15,16,17]. Thus, in summary, although new-onset AF is common in patients with acute PE, the potential role of AF as a contributing factor to PE requires further investigation.

Furthermore, the association between AF and deep vein thrombosis is less frequently discussed. Firstly, it is known that AF promotes blood stasis in the atria, resulting in thrombi [12]. Secondly, AF is associated with a pro-inflammatory state, which can influence the endothelial function of the vessels and thus promote clot formation [12,18]. However, AF itself does not directly lead directly to deep vein thrombosis [19], but there is evidence that the risk of deep vein thrombosis is increased, particularly in the first few months after AF has been diagnosed [17,20]. On the other hand, patients with AF often have other risk factors that contribute to the development of deep vein thrombosis, such as advanced age, limited mobility, or prolonged immobility due to hospitalization. The importance of comorbidities is also reflected in the significantly increased CHA_2_DS_2_-VA score in the PE cohort in this study. Notably, the control cohort was even older on average.

The relationship between advanced IAB and AF provides important insights into the pathophysiology of atrial arrhythmias and thromboembolic events [10,11]. The presence of IAB may also highlight underlying atrial structural remodeling as well as endothelial changes that are important for understanding the development of both AF and PE [6]. Atrial cardiomyopathy, which encompasses electrical, structural, and mechanical remodeling of the atria, plays a significant role in the pathophysiology of AF [5]. Electrical remodeling refers to alterations in the atrial conduction system, such as prolonged P-wave duration or the development of IAB [7]. Structural remodeling involves atrial dilation, fibrosis, and other changes that compromise normal atrial function, while mechanical remodeling refers to impaired atrial contraction, which may promote stasis and thrombus formation [6]. In addition, atrial cardiomyopathy is often associated with a hypercoagulable state and endothelial dysfunction, which increase the risk of thrombosis [18,21]. The presence of right atrial cardiomyopathy may add an additional layer of complexity in the formation of thrombi that may lead to PE [21]. It is plausible that right atrial cardiomyopathy, as part of the broader concept of atrial cardiomyopathy, could contribute to clot formation within the right atrium, thus increasing the risk of PE. The observation that advanced IAB was not associated with hemodynamic PE severity in our cohort suggests that advanced IAB may reflect chronic atrial abnormalities rather than acute right ventricular strain alone. This further strengthens the hypothesis that advanced IAB could serve as a stable ECG marker of atrial cardiomyopathy in patients at risk for thromboembolic complications.

Beyond these mechanistic considerations, it is important to emphasize that the rationale for conducting this study originated from a consistent clinical observation: a surprisingly large proportion of patients presenting with acute PE showed advanced IAB on their initial ECGs. In the absence of prior published evidence addressing this association, this prompted us to systematically evaluate the phenomenon in an exploratory manner. By documenting the prevalence and associated clinical features of advanced IAB in this context, the present study is intended to provide a foundation for future research and to stimulate further investigation into the electrophysiological and pathophysiological interplay between PE, atrial conduction disease, endothelial dysfunction and thromboembolic risk.

### Limitations

The present study has several limitations that should be acknowledged. First, the cohort size is relatively small, and the study was conducted at only one center, which may limit the generalizability of the findings. Additionally, the PE group was heterogeneous, including patients with varying severity and clinical presentations of PE. Another limitation is that only few patients with advanced IAB had previous ECGs available for comparison, so that a systemic evaluation of new IAB was not possible. Pre-PE ECGs were available for 30 patients, of whom 17 already exhibited interatrial block. The present study cannot establish a causal relationship regarding whether the advanced IAB was caused by the acute PE event. Furthermore, the study failed to detect an association between IAB and hemodynamic relevance, raising questions about the clinical significance of IAB (apart from AF) in this context.

## 5. Conclusions

The present study demonstrated for the first time that advanced IAB is a very frequent finding in patients with PE. The detection of advanced IAB on initial presentation to the emergency department may therefore provide an additional important marker of PE. The results highlight the significant association between advanced IAB and AF in patients with acute PE, which was not associated with right heart dysfunction. The relationship between atrial cardiomyopathy, IAB, AF, and PE should be analyzed in future research to better understand the mechanisms of thromboembolic events in these patients.

## Figures and Tables

**Table 1:** Comparison of patients with pulmonary embolism and control patients.

Variables	Patients with Pulmonary Artery Embolism (*n* = 351)	Control Patients (*n* = 120)	*p* Value
Age (years), mean (SD)	62.5 ± 17.1	67.9 ± 15.0	0.002
Women, *n* (%)	149 (42)	54 (45)	0.626
Body mass index (kg/m^2^), mean (SD)	27 ± 7	26 ± 5	0.205
CHA_2_DS_2_-Va Score (pts), mean (SD)	3.2 ± 2.9	1.6 ± 1.3	<0.001
New-onset AF, *n* (%)	29 (8)	0 (0)	<0.001
**Medical history**			
Heart failure, *n* (%)	118 (34)	0 (0)	<0.001
Arterial Hypertension, *n* (%)	195 (56)	56 (47)	0.092
Diabetes mellitus, *n* (%)	65 (19)	13 (11)	0.051
Stroke, *n* (%)	49 (14)	0 (0)	<0.001
Peripheral artery disease, *n* (%)	29 (8)	2 (2)	0.010
AF, *n* (%)	68 (19)	0 (0)	<0.001
Coronary artery disease, *n* (%)	71 (20)	1 (1)	<0.001
Myocardial infarction, *n* (%)	22 (6)	0 (0)	0.002
**Laboratory parameter**			
Creatinine (mg/dL), mean (SD)	1.2 ± 1.1	0.9 ± 0.2	0.002
eGFR (mL/min), mean (SD)	66 ± 23	83 ± 21	<0.001
TSH (mU/L), mean (SD)	2.8 ± 6.0	1.4 ± 1.5	0.019
Hemoglobin (g/dL), mean (SD)	12.4 ± 2.4	13.9 ± 1.5	<0.001
NT-proBNP (pg/mL), mean (SD)	3383 ± 6166	224 ± 312	<0.001
C-reactive protein (mg/dL), mean (SD)	5.5 ± 7.4	0.7 ± 2.5	<0.001
**Echocardiography**			
LVEF (%), mean (SD)	56 ± 9	57 ± 5	0.131
LA volume max (mL), mean (SD)	55 ± 26	46 ± 17	0.004
LA volume index (mL/m^2^), mean (SD)	28 ± 12	27 ± 9	0.433
RA volume max (mL), mean (SD)	36 ± 18	33 ± 15	0.529
RA volume index (mL/m^2^), mean (SD)	24 ± 13	17 ± 8	<0.001
RV diameter basal (mm), mean (SD)	39 ± 7	30 ± 5	<0.001
TAPSE (mm), mean (SD)	22 ± 5	28 ± 5	<0.001
Systolic pulmonary artery pressure (mmHg), mean (SD)	35 ± 15	17 ± 14	<0.001
Pulmonary hypertension, *n* (%)	77 (22)	9 (8)	<0.001
Mitral valve regurgitation, *n* (%)	91 (26)	20 (17)	<0.001
Tricuspid valve regurgitation, *n* (%)	141 (40)	60 (50)	0.270
**Electrocardiography**			
Heart rate (bpm), mean (SD)	88 ± 20	69 ± 12	<0.001
Sinus tachycardia, *n* (%)	82 (23)	0 (0)	<0.001
Advanced IAB, *n* (%)	122 (35)	2 (2)	<0.001
PR interval (ms), mean (SD)	175 ± 39	173 ± 29	0.678
P-wave duration (ms), mean (SD)	119 ± 18	117 ± 16	0.312
P-wave axis (°), mean (SD)	50 ± 24	50 ± 22	0.995
P-dextroatriale, *n* (%)	11 (3)	2 (2)	0.544
Right bundle branch block, *n* (%)	29 (8)	3 (3)	0.034
QRS right axis deviation, *n* (%)	57 (16)	19 (16)	0.917
S1Q3 pattern, *n* (%)	69 (20)	4 (3)	<0.001

Bpm, beats per minute; eGFR, estimated glomerular filtration rate; IAB, interatrial block; LA, left atrial; LVEF, left ventricular ejection fraction; RA, right atrial; RV, right ventricular; SD, standard deviation; TAPSE, tricuspid annular plane systolic excursion; TSH, thyroid stimulating hormone.

**Table 2:** Odds Ratios of the ECG parameters for pulmonary embolism.

ECG Parameters	Odds Ratio	Confidence Interval
Sinus tachycardia (>100 bpm)	1.305	1.232–1.382
P-dextroatriale	2.016	0.440–9.228
Advanced IAB	31.432	7.638–129.360
S1Q3 type	7.096	2.531–19.894
QRS right axis deviation	1.031	0.585–1.816
Right bundle branch block	3.557	1.063–11.896

Bpm, beats per minute; IAB, interatrial block.

**Table 3:** Comparison of patients with pulmonary embolism divided in patients with and without an advanced interatrial block on ECG.

	All Patients (*n* = 351)	Patients with IAB (*n* = 122)	Patients Without IAB (*n* = 229)	*p* Value *
Age (years), mean (SD)	62.5 ± 17.2	69.7 ± 15.3	58.6 ± 16.9	<0.001
Women, *n* (%)	147 (42)	47 (39)	100 (44)	0.352
BMI (kg/m^2^), mean (SD)	27.1 ± 7.4	28.1 ± 7.8	26.6 ± 7.1	0.128
CHA_2_DS_2_-Va Score (pts), mean (SD)	3.2 ± 3.0	4.3 ± 3.0	2.7 ± 2.8	<0.001
ECMO, *n* (%)	15 (4)	5 (4)	10 (4)	0.918
Death, *n* (%)	31 (9)	13 (11)	19 (8)	0.465
New-onset AF, *n* (%)	29 (8)	19 (16)	10 (4)	<0.001
**Medical history**				
Heart failure, *n* (%)	118 (34)	46 (38)	72 (31)	0.237
Arterial Hypertension, *n* (%)	195 (56)	84 (69)	111 (48)	<0.001
Diabetes mellitus, *n* (%)	65 (19)	27 (22)	38 (17)	0.203
Stroke, *n* (%)	49 (14)	15 (12)	34 (15)	0.511
Peripheral artery disease, *n* (%)	29 (8)	8 (7)	21 (9)	0.397
Malignant disease, *n* (%)	127 (36)	37 (30)	90 (39)	0.096
AF, *n* (%)	68 (19)	36 (30)	32 (14)	<0.001
Coronary artery disease, *n* (%)	71 (20)	36 (30)	35 (15)	0.002
Myocardial infarction, *n* (%)	22 (6)	10 (8)	12 (5)	0.276
**Laboratory parameter**				
Creatinine (mg/dL), mean (SD)	1.2 ± 1.1	1.4 ± 1.7	1.1 ± 0.6	0.018
eGFR (mL/min), mean (SD)	66 ± 23	61 ± 23	69 ± 22	0.001
TSH (mU/L), mean (SD)	2.8 ± 6.0	2.8 ± 5.3	2.8 ± 6.4	0.995
Hemoglobin (g/dL), mean (SD)	12.4 ± 2.4	12.7 ± 2.4	12.3 ± 2.4	0.094
CRP (mg/dL), mean (SD)	5.5 ± 7.4	5.6 ± 7.6	5.4 ± 7.3	0.809
NT-proBNP (pg/mL), mean (SD)	3383 ± 6166	4166 ± 7115	2928 ± 5518	0.156
D-dimer (μg/mL), mean (SD)	7.6 ± 6.6	7.8 ± 6.5	7.5 ± 6.7	0.778
Troponin T hs (ng/L), mean (SD)	112 ± 351	115 ± 364	111 ± 344	0.943
**Echocardiography**				
LVEF (%), mean (SD)	56 ± 9	56 ± 10	56 ± 9	0.689
LA volume (mL), mean (SD)	55 ± 26	61 ± 28	52 ± 24	0.072
LA volume index (mL/m^2^), mean (SD)	28 ± 12	32 ± 14	27 ± 11	0.033
RA volume (mL), mean (SD)	36 ± 18	36 ± 21	36 ± 16	0.986
RA volume index (mL/m^2^), mean (SD)	24 ± 13	26 ± 14	23 ± 12	0.195
RV diameter basal (mm), mean (SD)	39 ± 7	39 ± 7	39 ± 7	0.929
TAPSE (mm), mean (SD)	22 ± 5	21 ± 6	22 ± 5	0.211
sPAP (mmHg), mean (SD)	35 ± 15	34 ± 13	35 ± 16	0.809
Pulmonary hypertension, *n* (%)	77 (22)	32 (26)	45 (20)	0.260
Mitral valve regurgitation, *n* (%)	91 (26)	43 (35)	48 (21)	0.002
Tricuspid valve regurgitation, *n* (%)	141 (40)	58 (48)	83 (36)	0.022
**Electrocardiography**				
Heart rate (bpm), mean (SD)	88 ± 20	83 ± 18	91 ± 20	<0.001
Sinus tachycardia, *n* (%)	82 (23)	20 (16)	62 (27)	0.024
PR interval (ms), mean (SD)	175 ± 39	196 ± 44	164 ± 31	<0.001
P-wave duration (ms), mean (SD)	119 ± 18	131 ± 15	112 ± 16	<0.001
P dextroatriale, *n* (%)	11 (3)	1 (1)	10 (3)	0.076
P-wave axis (°), mean (SD)	50 ± 24	44 ± 28	54 ± 20	<0.001
S1Q3 pattern, *n* (%)	69 (20)	19 (16)	50 (22)	0.160
QRS right axis deviation, *n* (%)	57 (16)	10 (8)	47 (21)	0.003
Right bundle branch block, *n* (%)	29 (8)	7 (6)	22 (10)	0.217

* for patients with and without IAB; BMI, body mass index; bpm, beats per minute; CRP, C-reactive protein; ECMO, extracorporeal membrane oxygenation; eGFR, estimated glomerular filtration rate; IAB, interatrial block; LA, left atrial; LVEF, left ventricular ejection fraction; RA, right atrial; RV, right ventricular; SD, standard deviation; sPAP, systolic pulmonary artery pressure; TAPSE, tricuspid annular plane systolic excursion; TSH, thyroid stimulating hormone.

## Data Availability

The raw data supporting the conclusions of this article will be made available by the authors on request.
